# Supplementary material for: Rhizocompartmental microbiomes of arrow bamboo (Fargesia nitida) and their relation to soil properties in Subalpine Coniferous Forests
Source: PeerJ. 2023 Nov 29;11:e16488. doi: 10.7717/peerj.16488 (PMC10693234; doi:10.7717/peerj.16488)
Supplement: Supplemental Information 1 [file peerj-11-16488-s001.docx]

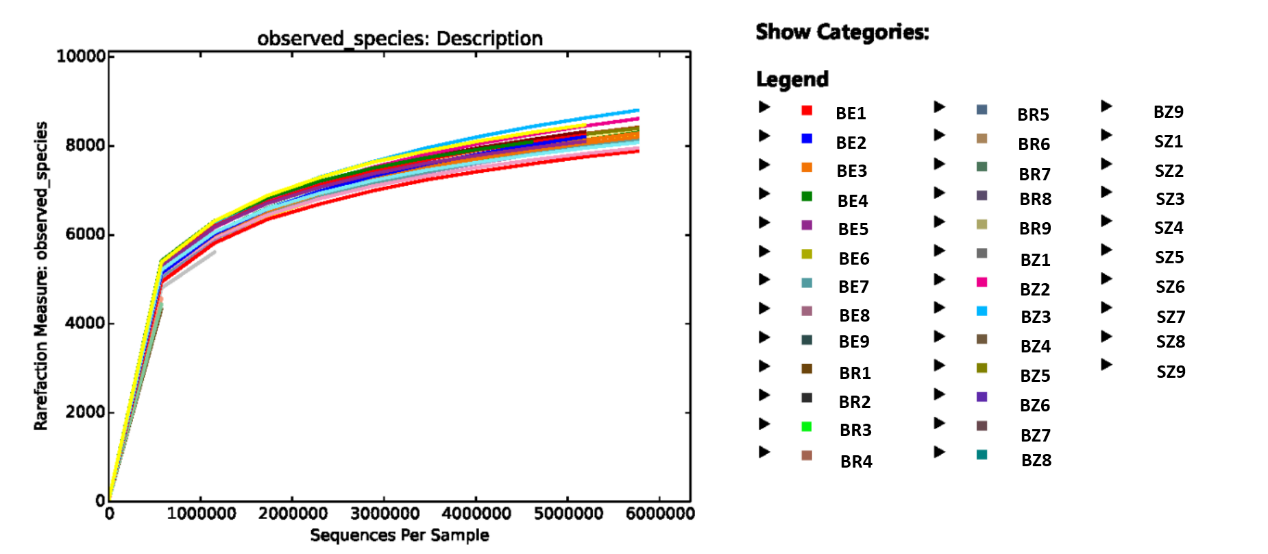


Fig. S1 Rarefaction analysis of observed species.


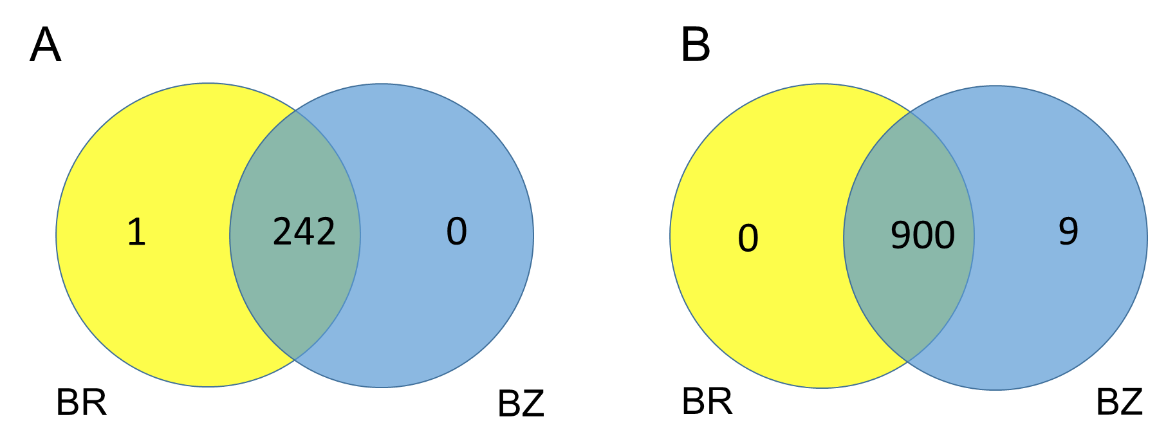


Fig. S2 Venn diagrams of the significantly enriched (a) and depleted (b) genus of the microbiome colonized in root compared with rhizosphere and root zone soil.


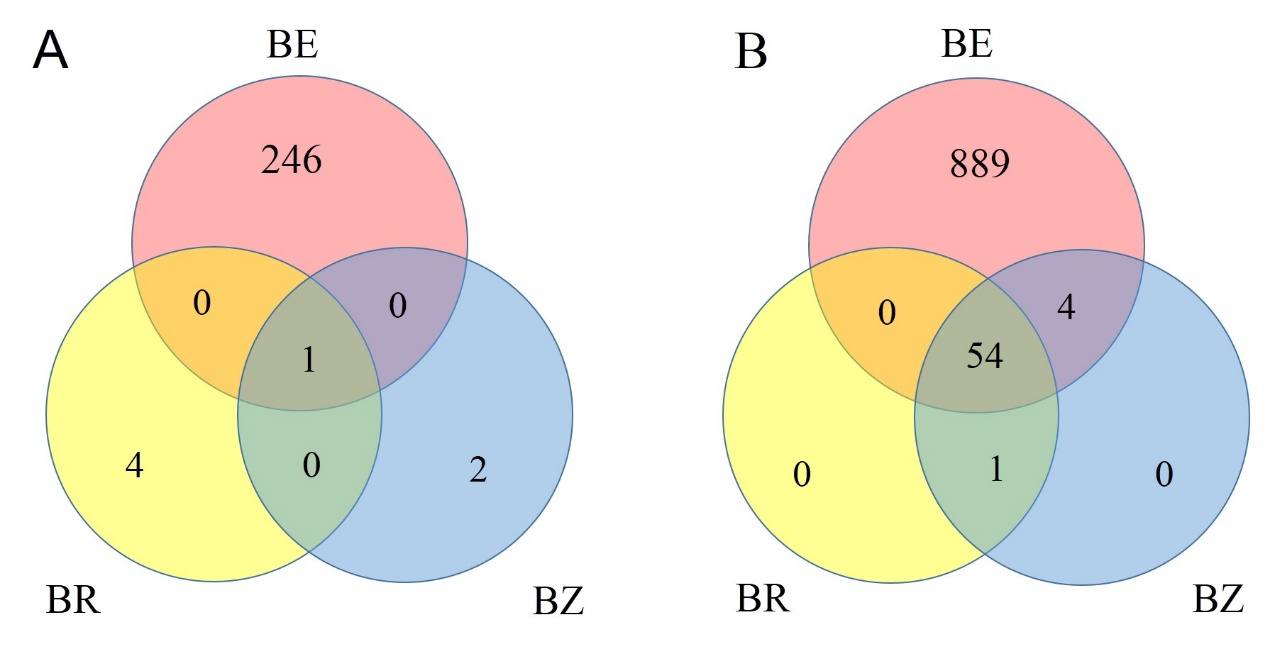


Fig S3. Venn diagrams of the significantly enriched (a) and depleted (b) genus of the microbiome colonized in the arrow bamboo roots and soils compared with spruce root zone soils. BE: arrow bamboo root endosphere, BR: arrow bamboo rhizosphere, BZ: arrow bamboo root zone.


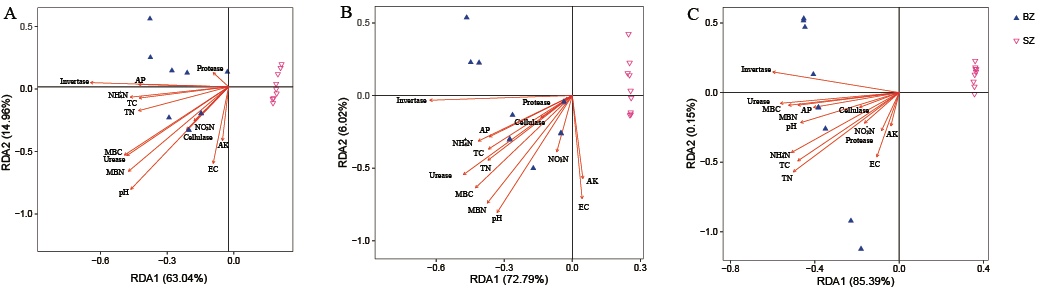


Fig S4. Redundancy analysis (RDA) of soil taxonomic (A: the genus level) and functional (B: KEGG pathway level and C: CAZy level) structure of microbiomes and environmental factors (B).

**Supplementary Table S1** Summary of the metagenome sequencing of 36 samples of roots and soils

| Samples | Raw reads | Raw bases (bp) | High quality  Reads (%) | High quality  Data (%) | Contigs | N50(bp) | N90(bp) | Min(bp) | Max(bp) | N50(bp) |
| --- | --- | --- | --- | --- | --- | --- | --- | --- | --- | --- |
| BE -1 | 119209368 | 17881405200 | 99 | 98.94 | 918694 | 1062 | 393 | 200 | 36571 | 1062 |
| BE-2 | 116636034 | 17495405100 | 99.06 | 99 | 1081920 | 907 | 373 | 200 | 36571 | 907 |
| BE-3 | 112887964 | 16933194600 | 99.04 | 98.97 | 980766 | 936 | 380 | 200 | 54090 | 936 |
| BE-4 | 117730234 | 17659535100 | 99.12 | 99.05 | 1137539 | 819 | 363 | 200 | 46745 | 819 |
| BE-5 | 111887366 | 16783104900 | 99.05 | 98.98 | 973154 | 924 | 381 | 200 | 50366 | 924 |
| BE-6 | 107130304 | 16069545600 | 99.23 | 99.16 | 1272169 | 823 | 363 | 200 | 1101311 | 823 |
| BE-7 | 108074544 | 16211181600 | 98.91 | 98.83 | 892089 | 999 | 395 | 200 | 27624 | 999 |
| BE-8 | 110483042 | 16572456300 | 98.98 | 98.92 | 901446 | 997 | 394 | 200 | 31938 | 997 |
| BE-9 | 104185860 | 15627879000 | 99.01 | 98.96 | 848446 | 1029 | 403 | 200 | 36450 | 1029 |
| BR-1 | 75271274 | 11290691100 | 99.71 | 99.66 | 1531061 | 397 | 316 | 200 | 19115 | 397 |
| BR-2 | 77523954 | 11628593100 | 99.62 | 99.6 | 1307133 | 414 | 319 | 200 | 9503 | 414 |
| BR-3 | 71941966 | 10791294900 | 99.51 | 99.49 | 1405109 | 470 | 322 | 200 | 20153 | 470 |
| BR-4 | 71498440 | 10724766000 | 99.43 | 99.41 | 1499579 | 451 | 321 | 200 | 58404 | 451 |
| BR-5 | 69317526 | 10397628900 | 99.65 | 99.61 | 1899340 | 560 | 335 | 200 | 1187338 | 560 |
| BR-6 | 73652454 | 11047868100 | 99.57 | 99.54 | 2021794 | 551 | 332 | 200 | 281613 | 551 |
| BR-7 | 78624114 | 11793617100 | 99.67 | 99.63 | 2408779 | 441 | 323 | 200 | 87448 | 441 |
| BR-8 | 71456828 | 10718524200 | 99.67 | 99.63 | 1550993 | 438 | 321 | 200 | 41105 | 438 |
| BR-9 | 67036268 | 10055440200 | 99.65 | 99.61 | 1471099 | 437 | 321 | 200 | 27617 | 437 |
| BZ-1 | 81719718 | 12257957700 | 99.61 | 99.58 | 1567931 | 422 | 319 | 200 | 52156 | 422 |
| BZ-2 | 89290108 | 13393516200 | 99.65 | 99.63 | 1540650 | 411 | 318 | 200 | 28795 | 411 |
| BZ-3 | 92190876 | 13828631400 | 99.6 | 99.58 | 1926829 | 442 | 322 | 200 | 10554 | 442 |
| BZ-4 | 75609548 | 11341432200 | 99.67 | 99.66 | 1621046 | 441 | 321 | 200 | 55498 | 441 |
| BZ-5 | 81722592 | 12258388800 | 99.58 | 99.54 | 2288880 | 546 | 336 | 200 | 1032642 | 546 |
| BZ-6 | 77657302 | 11648595300 | 99.57 | 99.53 | 1976434 | 539 | 332 | 200 | 656925 | 539 |
| BZ-7 | 83171018 | 12475652700 | 99.62 | 99.55 | 2251056 | 504 | 330 | 200 | 171484 | 504 |
| BZ-8 | 75987500 | 11398125000 | 99.67 | 99.63 | 1777135 | 477 | 326 | 200 | 28344 | 477 |
| BZ-9 | 67721394 | 10158209100 | 99.75 | 99.73 | 1671320 | 469 | 325 | 200 | 13057 | 469 |
| SZ-1 | 68590300 | 10288545000 | 99.66 | 99.63 | 1805712 | 476 | 327 | 200 | 287749 | 476 |
| SZ-2 | 66974106 | 10046115900 | 99.59 | 99.58 | 1483518 | 477 | 324 | 200 | 21046 | 477 |
| SZ-3 | 70147952 | 10522192800 | 99.69 | 99.66 | 1616808 | 493 | 327 | 200 | 149670 | 493 |
| SZ-4 | 72709942 | 10906491300 | 99.69 | 99.61 | 1976037 | 567 | 334 | 200 | 192473 | 567 |
| SZ-5 | 94064864 | 14109729600 | 99.6 | 99.58 | 2151320 | 566 | 338 | 200 | 829990 | 566 |
| SZ-6 | 81278220 | 12191733000 | 99.49 | 99.47 | 2162975 | 604 | 339 | 200 | 1157486 | 604 |
| SZ-7 | 83645974 | 12546896100 | 99.61 | 99.58 | 2276089 | 547 | 337 | 200 | 98656 | 547 |
| SZ-8 | 83743654 | 12561548100 | 99.63 | 99.6 | 2020890 | 504 | 330 | 200 | 47114 | 504 |
| SZ-9 | 74891094 | 11233664100 | 99.71 | 99.67 | 1692226 | 474 | 325 | 200 | 114764 | 474 |

BE: bamboo root endosphere, BR: bamboo rhizosphere, BZ: bamboo root zone, SZ: spruce root zone.

**Supplementary Table S2** Comparative analysis of the relative abundance (%) of Archaea, Bacteria, and Eukarya in the roots and soils.

| Domain | BE | BR | BZ | SZ |
| --- | --- | --- | --- | --- |
| Archaea | 0.07±0.01b | 0.19±0.03a | 0.20±0.02a | 0.27±0.04 |
| Bacteria | 74.45±4.80b | 99.37±0.04a | 99.39±0.04a | 99.13±0.19 |
| Eukaryota | 25.46±4.80a | 0.43±0.03b | 0.40±0.04b | 0.58±0.19 |
| Viruses | 0.02±0.00a | 0.01±0.00a | 0.01±0.00a | 0.02±0.00* |
| **Total** | 100% | 100% | 100% | 100% |

Different lowercase letters represent significant differences among bamboo samples using Duncan's multiple range test. T test was used for comparing root zone soil of bamboo and spruce **p* < .05. BE: bamboo root endosphere, BR: bamboo rhizosphere, BZ: bamboo root zone, SZ: spruce root zone.

**Supplementary Table S3** Comparative analysis of the dominant phyla relative abundance of Archaea, Bacteria, and Eukarya in the roots and soils.

| Domain | phyla | BE | BR | BZ | SZ |
| --- | --- | --- | --- | --- | --- |
| Bacteria | Proteobacteria | 5.98E-01b | 7.18E-01a | 7.16E-01a | 6.87E-01 |
|  | Actinobacteria | 6.45E-02b | 1.12E-01a | 1.10E-01a | 1.30E-01 |
|  | Planctomycetes | 1.20E-02b | 1.67E-02a | 1.69E-02a | 1.74E-02 |
|  | Acidobacteria | 1.90E-02b | 4.44E-02a | 4.72E-02a | 5.03E-02 |
|  | Bacteroidetes | 7.24E-03b | 1.38E-02a | 1.37E-02a | 1.20E-02 |
|  | Verrucomicrobia | 4.22E-03b | 1.10E-02a | 1.08E-02a | 9.93E-03 |
|  | Gemmatimonadetes | 2.19E-03b | 6.42E-03a | 6.62E-03a | 6.60E-03 |
|  | Firmicutes | 2.36E-03b | 5.59E-03a | 5.85E-03a | 7.40E-03 |
|  | Cyanobacteria | 9.33E-04a | 2.17E-03b | 2.27E-03b | 2.51E-03 |
|  | Chloroflexi | 8.27E-04a | 1.81E-03b | 1.89E-03b | 2.13E-03 |
|  | Deinococcus-Thermus | 8.53E-04b | 2.01E-03a | 2.10E-03a | 2.28E-03 |
|  | Nitrospirae | 6.02E-04b | 1.95E-03a | 2.05E-03a | 1.91E-03 |
|  | Chlorobi | 2.61E-04a | 6.29E-04b | 6.64E-04b | 7.40E-04 |
|  | Armatimonadetes | 1.98E-04b | 4.85E-04a | 4.91E-04a | 5.14E-04 |
|  | Tenericutes | 1.91E-04a | 4.12E-05b | 3.60E-05b | 3.38E-05 |
|  | Kiritimatiellaeota | 1.59E-04a | 1.41E-04a | 1.44E-04a | 1.58E-04 |
|  | Chlamydiae | 1.52E-04a | 1.62E-04a | 1.62E-04a | 1.53E-04 |
|  | Spirochaetes | 1.21E-04b | 2.60E-04a | 2.72E-04a | 2.89E-04 |
|  | Lentisphaerae | 7.36E-05b | 1.99E-04a | 2.00E-04a | 2.21E-04 |
|  | Synergistetes | 4.35E-05b | 1.15E-04a | 1.22E-04a | 1.30E-04 |
|  | Aquificae | 2.65E-05b | 5.86E-05a | 6.05E-05a | 6.94E-05* |
|  | Calditrichaeota | 2.80E-05b | 7.08E-05a | 7.61E-05a | 8.83E-05 |
|  | Thermotogae | 2.03E-05c | 5.17E-05b | 5.47E-05b | 6.34E-05* Aquificae |
|  | Ignavibacteriae | 1.81E-05b | 4.39E-05a | 4.58E-05a | 4.93E-05 |
|  | Chrysiogenetes | 1.26E-05b | 3.81E-05a | 3.99E-05a | 4.42E-05 |
|  | Elusimicrobia | 8.91E-06b | 1.97E-05a | 1.99E-05a | 2.18E-06 |
|  | Fusobacteria | 6.45E-06b | 1.65E-05a | 1.67E-05a | 1.83E-05 |
|  | Thermodesulfobacteria | 6.62E-06c | 1.68E-05 | 1.87E-05b | 2.36E-05* |
|  | Fibrobacteres | 4.92E-06b | 1.29E-05a | 1.32E-05a | 1.44E-05 |
|  | Deferribacteres | 3.39E-06c | 1.20E-05b | 1.24E-05 | 1.69E-05* |
|  | Coprothermobacterota | 1.84E-06b | 5.02E-06a | 5.30E-06a | 5.31E-06 |
|  | Dictyoglomi | 1.35E-06b | 6.49E-06a | 7.18E-06a | 8.51E-06 |
|  | Caldiserica | 9.40E-07a | 1.94E-06a | 1.80E-06a | 1.77E-06 |
|  | Nitrospinae | 5.16E-07a | 5.43E-07a | 4.88E-07a | 5.43E-07 |
| Eukaryota | Ascomycota | 3.12E-02a | 1.64E-03b | 1.58E-03b | 1.80E-03 |
|  | Basidiomycota | 1.21E-02a | 8.50E-04b | 8.17E-04b | 1.01E-03 |
|  | Mucoromycota | 8.68E-03a | 2.50E-04b | 1.65E-04b | 4.92E-05** |
|  | Zoopagomycota | 1.82E-03a | 9.50E-07b | 5.80E-07b | 5.20E-07 |
|  | Apicomplexa | 1.22E-03a | 6.89E-05b | 5.85E-05b | 2.28E-04 |
|  | Bacillariophyta | 6.66E-05a | 9.48E-06b | 9.67E-06b | 9.11E-06 |
|  | Euglenida | 3.05E-05a | 9.44E-06b | 9.50E-06b | 1.06E-05 |
|  | Microsporidia | 1.31E-05a | 1.37E-06b | 1.61E-06b | 1.35E-06 |
|  | Chytridiomycota | 8.64E-06a | 3.31E-06b | 3.63E-06b | 4.21E-06 |
|  | Blastocladiomycota | 8.90E-07a | 9.90E-07a | 1.24E-06a | 1.24E-06 |
|  | Chromerida | 3.70E-07a | 6.10E-07a | 7.00E-07a | 5.00E-07 |
|  | Olpidiomycota | 0.00E+00a | 0.00E+00a | 0.00E+00a | 1.50E-07 |
| Archaea | Euryarchaeota | 5.05E-04b | 1.22E-03a | 1.28E-03a | 1.38E-03 |
|  | Thaumarchaeota | 1.08E-04c | 5.62E-04b | 5.84E-04b | 1.20E-03 |
|  | Crenarchaeota | 1.28E-05c | 2.50E-05b | 2.88E-05ab | 3.30E-05 |
|  | Nanoarchaeota | 1.10E-07a | 2.30E-07a | 3.20E-07a | 1.20E-07 |

Different lowercase letters represent significant differences among bamboo samples using Duncan's multiple range test. T test was used for comparing root zone soil of bamboo and spruce **p* < .05. BE: bamboo root endosphere, BR: bamboo rhizosphere, BZ: bamboo root zone, SZ: spruce root zone.

**Supplementary Table S4** PERMANOVA analysis (global test and pairwise comparison) of microbial communities (genus), and the gene families (KEGG level 3 and CAZy level 2) in root and soils of bamboo and spruce.

|  | Global test | | | Pairwise comparison | | | |
| --- | --- | --- | --- | --- | --- | --- | --- |
|  | F | R^2^ | P |  | F | R^2^ | P |
| Microbial communities | 318.43 | 0.968 | 0.001 | BE vs BR | 23.775 | 0.598 | **0.001** |
|  |  |  |  | BE vs BZ | 24.804 | 0.608 | **0.001** |
|  |  |  |  | BE vs SZ | 24.719 | 0.607 | **0.001** |
|  |  |  |  | BR vs BZ | 0.981 | 0.006 | 0.981 |
|  |  |  |  | BR vs SZ | 0.981 | 0.058 | 0.378 |
|  |  |  |  | BZ vs SZ | 0.799 | 0.048 | 0.468 |
| KEGG genes | 31.246 | 0.746 | 0.001 | BE vs BR | 34.072 | 0.680 | **0.001** |
|  |  |  |  | BE vs BZ | 34.257 | 0.682 | **0.001** |
|  |  |  |  | BE vs SZ | 34.440 | 0.683 | **0.001** |
|  |  |  |  | BR vs BZ | 0.051 | 0.003 | 0.992 |
|  |  |  |  | BR vs SZ | 1.020 | 0.060 | 0.405 |
|  |  |  |  | BZ vs SZ | 0.714 | 0.043 | 0.525 |
| CAZy genes | 318.430 | 0.968 | 0.001 | BE vs BR | 397.426 | 0.961 | **0.001** |
|  |  |  |  | BE vs BZ | 394.646 | 0.961 | **0.001** |
|  |  |  |  | BE vs SZ | 392.889 | 0.961 | **0.001** |
|  |  |  |  | BR vs BZ | 0.160 | 0.061 | 0.946 |
|  |  |  |  | BR vs SZ | 1.031 | 0.061 | 0.335 |
|  |  |  |  | BZ vs SZ | 0.637 | 0.038 | 0.525 |

BE: arrow bamboo root endosphere, BR: arrow bamboo rhizosphere, BZ: arrow bamboo root zone, SZ: spruce root zone.

Table S5 Selected genes related to C and N cycling and their abundances in the roots and soils.

| Function group |  | BE | BR | BZ | SZ | KO_descriptionon |
| --- | --- | --- | --- | --- | --- | --- |
| Nitrogen Fixation | K02584 | 8.93E-05b | 2.22E-04a | 2.22E-04a | 2.22E-04 | Nif-specific regulatory protein |
|  | K02585 | 2.42E-06a | 2.20E-07b | 2.40E-07b | 5.40E-07 | nitrogen fixation protein NifB |
|  | K02586 | 1.34E-06a | 3.20E-07b | 2.20E-07b | 7.10E-07 | nitrogenase molybdenum-iron protein alpha chain |
|  | K02587 | 7.50E-07a | 5.20E-07a | 4.30E-07a | 1.02E-06 | nitrogenase molybdenum-cofactor synthesis protein NifE |
|  | K02588 | 1.14E-06a | 3.50E-07a | 3.90E-07a | 9.10E-07 | nitrogenase iron protein NifH |
|  | K02589 | - | 2.30E-07a | 1.60E-07a | 1.10E-07 | nitrogen regulatory protein PII 1 |
|  | K02590 | 5.60E-07a | 1.00E-07a | 1.70E-07a | 1.50E-07 | nitrogen regulatory protein PII 2 |
|  | K02591 | 1.57E-06a | 2.20E-07a | 2.50E-07a | 8.00E-07 | nitrogenase molybdenum-iron protein beta chain |
|  | K02592 | 1.20E-07a | 4.90E-07a | 7.40E-07a | 1.19E-06 | nitrogenase molybdenum-iron protein NifN |
|  | K02593 | - | 1.50E-07a | 1.10E-07a | 2.70E-07 | nitrogen fixation protein NifT |
|  | K02594 | 4.37E-06b | 9.36E-06a | 9.39E-06a | 1.34E-05 | homocitrate synthase NifV |
|  | K02597 | 5.00E-07a | 2.40E-07a | 2.20E-07a | 3.00E-07 | nitrogen fixation protein NifZ |
|  | K02598 | 6.87E-06b | 1.15E-05a | 1.12E-05a | 1.41E-05 | nitrite transporter NirC |
| Nitrate Reduction | K00368 | 5.54E-05a | 6.78E-05a | 6.94E-05a | 8.04E-05 | nitrite reductase (NO-forming) |
|  | K00369 | 7.44E-05a | 4.44E-05a | 4.36E-05a | 6.43E-05 | nitrate reductase-like protein |
|  | K00370 | 2.54E-05a | 1.69E-05a | 1.51E-05a | 2.33E-05* | nitrate reductase 1, alpha subunit |
|  | K00371 | 4.29E-04a | 4.19E-04a | 4.16E-04a | 3.83E-04* | nitrate reductase 1, beta subunit |
|  | K00372 | 9.67E-06a | 6.92E-06a | 7.01E-06a | 9.85E-06 | nitrate reductase catalytic subunit |
|  | K00373 | 9.60E-06a | 1.01E-05a | 9.71E-06a | 1.45E-05 | nitrate reductase 1, delta subunit |
|  | K00374 | 9.61E-06a | 1.01E-05a | 9.71E-06a | 1.45E-05* | nitrate reductase 1, gamma subunit |
| Denitrification | K00376 | 8.87E-06b | 1.67E-05a | 1.95E-05a | 2.46E-05 | nitrous-oxide reductase |
|  | K07218 | 5.81E-06b | 1.01E-05a | 1.01E-05a | 1.56E-05 | nitrous oxidase accessory protein |
|  | K04561 | 8.88E-05b | 1.21E-04ab | 1.30E-04a | 1.62E-04 | nitric oxide reductase subunit B |
| Ammonification | K00360 | 2.09E-06a | 1.35E-06a | 1.61E-06a | 1.86E-06 | nitrate reductase (NADH) |
|  | K00362 | 4.51E-04a | 3.35E-04b | 3.22E-04b | 2.94E-04 | nitrite reductase (NAD(P)H) large subunit |
|  | K00363 | 5.29E-05a | 6.32E-05a | 6.46E-05a | 6.89E-05 | nitrite reductase (NAD(P)H) small subunit |
|  | K00366 | 2.78E-04a | 2.10E-04b | 2.06E-04b | 2.08E-04 | ferredoxin-nitrite reductase |
|  | K00367 | 2.33E-05a | 3.32E-05a | 3.37E-05a | 3.10E-05 | ferredoxin-nitrate reductase |
|  | K03385 | 1.14E-05a | 2.92E-05a | 2.96E-05a | 2.81E-05 | cytochrome c-552 |
| NADH Dehydrogenase I | K00329 | 9.7E-05b | 1.45E-04a | 1.42E-04a | 1.45E-04 | NADH dehydrogenase |
|  | K00330 | 1.33E-04b | 2.73E-04a | 2.67E-04a | 2.66E-04 | NADH-quinone oxidoreductase subunit A |
|  | K00331 | 2.15E-04b | 2.69E-04a | 2.69E-04a | 2.72E-04 | NADH-quinone oxidoreductase subunit B |
|  | K00332 | 2.27E-04b | 4.26E-04a | 4.27E-04a | 4.32E-04 | NADH-quinone oxidoreductase subunit C |
|  | K00333 | 4.28E-04b | 6.85E-04a | 6.87E-04a | 6.77E-04 | NADH-quinone oxidoreductase subunit D |
|  | K00334 | 1.98E-04b | 3.18E-04a | 3.20E-04a | 3.16E-04 | NADH-quinone oxidoreductase subunit E |
|  | K00335 | 4.31E-04b | 7.22E-04a | 7.31E-04a | 7.27E-04 | NADH-quinone oxidoreductase subunit F |
|  | K00336 | 6.36E-04b | 8.64E-04a | 8.58E-04a | 8.50E-04 | NADH-quinone oxidoreductase subunit G |
|  | K00337 | 4.31E-04b | 7.71E-04a | 7.82E-04a | 7.72E-04 | NADH-quinone oxidoreductase subunit H |
|  | K00338 | 1.70E-04b | 2.94E-04a | 2.97E-04a | 2.93E-04 | NADH-quinone oxidoreductase subunit I |
|  | K00339 | 2.11E-04b | 3.93E-04a | 3.95E-04a | 4.04E-04 | NADH-quinone oxidoreductase subunit J |
|  | K00340 | 9.48E-05b | 1.85E-04a | 1.87E-04a | 1.80E-04 | NADH-quinone oxidoreductase subunit K |
|  | K00341 | 9.34E-04b | 1.38E-03a | 1.36E-03a | 1.36E-03 | NADH-quinone oxidoreductase subunit L |
|  | K00342 | 6.27E-04b | 1.11E-03a | 1.11E-03a | 1.11E-03 | NADH-quinone oxidoreductase subunit M |
|  | K00343 | 5.51E-04b | 9.43E-04a | 9.45E-04a | 9.42E-04 | NADH-quinone oxidoreductase subunit N |
| Pyruvate Dehydrogenase E1 & E2 | K00156 | 3.83E-04a | 4.52E-04a | 4.68E-04a | 4.69E-04 | pyruvate dehydrogenase (quinone) |
|  | K00158 | 7.57E-06b | 1.36E-05a | 1.50E-05a | 1.77E-05 | pyruvate oxidase |
|  | K00161 | 5.22E-04b | 7.41E-04a | 7.42E-04a | 7.69E-04 | pyruvate dehydrogenase E1 component subunit alpha |
|  | K00162 | 4.31E-04b | 6.31E-04a | 6.25E-04a | 6.43E-04 | pyruvate dehydrogenase E1 component subunit beta |
|  | K00163 | 7.23E-04b | 9.21E-04a | 9.07E-04a | 8.78E-04 | pyruvate dehydrogenase E1 component |
|  | K00627 | 2.43E-03a | 9.51E-04b | 9.44E-04b | 9.70E-04 | pyruvate dehydrogenase E2 component (dihydrolipoamide |
|  |  |  |  |  |  | acetyltransferase) |
|  | K00898 | 3.77E-06a | 5.50E-07ab | 5.10E-07ab | 2.70E-07 | pyruvate dehydrogenase kinase |
| Pyruvate Ferridoxin Oxidoreductase | K00169 | 5.36E-06b | 1.69E-05a | 1.80E-05a | 2.37E-05 | pyruvate ferredoxin oxidoreductase, alpha subunit |
|  | K00170 | 7.63E-06a | 1.17E-05a | 1.31E-05a | 1.92E-05 | pyruvate ferredoxin oxidoreductase, beta subunit |
|  | K00171 | 2.85E-06b | 6.64E-06a | 8.30E-06a | 8.69E-06 | pyruvate ferredoxin oxidoreductase, delta subunit |
|  | K00172 | 1.74E-06b | 3.35E-06ab | 4.02E-06a | 5.73E-06 | pyruvate ferredoxin oxidoreductase, gamma subunit |
| Cellulose Degradation | K05349 | 1.94E-03a | 1.84E-03a | 1.83E-03a | 1.81E-03 | beta-glucosidase |
|  | K05350 | 5.67E-04a | 5.07E-04b | 4.91E-04b | 5.13E-04 | beta-glucosidase |
|  | K01187 | 6.55E-04b | 8.70E-04a | 8.72E-04a | 9.10E-04 | alpha-glucosidase |
|  | K01188 | 2.52E-04a | 9.60E-07b | 9.50E-07b | 1.34E-06 | beta-glucosidase |
|  | K01222 | 4.66E-05b | 6.77E-05a | 6.75E-05a | 8.83E-05 | 6-phospho-beta-glucosidase |
|  | K01223 | 1.86E-05a | 1.98E-06b | 2.66E-06b | 2.51E-06 | 6-phospho-beta-glucosidase |
|  | K01179 | 5.39E-04a | 5.44E-04a | 5.36E-04a | 5.64E-04 | endoglucanase |
|  | K00702 | 2.86E-05a | 3.50E-05a | 3.21E-05a | 3.20E-05 | cellobiose phosphorylase |
| Cellobiose Transport | K10240 | 6.44E-06a | 7.55E-06a | 7.07E-06a | 5.63E-06 | cellobiose transport system substrate-binding protein |
|  | K10241 | 7.56E-06a | 6.51E-06a | 6.61E-06a | 5.52E-06 | cellobiose transport system permease protein |
|  | K10242 | 7.59E-06a | 5.25E-06a | 5.13E-06a | 4.48E-06 | cellobiose transport system permease protein |
|  | K01225 | 5.51E-06a | 1.30E-07c | 1.40E-07c | 6.40E-07 | cellulose 1,4-beta-cellobiosidase |
| Hemi-cellulose Degradation | K01218 | 3.11E-05b | 5.27E-05a | 4.99E-05a | 5.07E-05 | mannan endo-1,4-beta-mannosidase |
|  | K01805 | 2.54E-04a | 3.06E-04a | 3.01E-04a | 2.86E-04 | xylose isomerase |
| Chitin Degradation | K03791 | 3.67E-05a | 3.16E-05a | 3.23E-05a | 3.36E-05 | putative chitinase |
|  | K01183 | 6.86E-04a | 2.13E-04b | 2.13E-04b | 2.21E-04 | chitinase |
|  | K01452 | - | 5.20E-07a | 4.90E-07a | 4.90E-07 | chitin deacetylase |
| Sugar Utilization | K01804 | 1.24E-04a | 1.79E-04a | 1.78E-04a | 1.77E-04 | L-arabinose isomerase |
|  | K01805 | 2.54E-04b | 3.06E-04a | 3.01E-04a | 2.86E-04 | xylose isomerase |
|  | K00845 | 3.87E-04b | 6.43E-04a | 6.44E-04a | 7.08E-04 | glucokinase |
|  | K00847 | 3.52E-04a | 2.84E-04b | 2.88E-04b | 3.02E-04 | fructokinase |
|  | K00849 | 1.35E-04b | 2.43E-04a | 2.47E-04a | 2.65E-04 | galactokinase |
|  | K00886 | 1.66E-04b | 2.76E-04a | 2.84E-04a | 3.11E-04 | polyphosphate glucokinase |
|  | K00854 | 4.87E-04a | 5.15E-04a | 5.08E-04a | 5.32E-04 | xylulokinase |
|  | K12308 | 3.01E-04a | 3.41E-04a | 3.40E-04a | 3.58E-04 | beta-galactosidase |
|  | K02793 | 3.06E-05b | 5.64E-05a | 5.65E-05a | 4.70E-05 | PTS system, mannose-specific IIA component |
| Sugar Transporters | K10188 | 9.74E-06a | 6.75E-06a | 6.37E-06a | 5.87E-06 | lactose/L-arabinose transport system substrate-binding protein |
|  | K10189 | 7.42E-06a | 6.34E-06a | 6.29E-06a | 4.51E-06 | lactose/L-arabinose transport system permease protein |
|  | K10190 | 1.02E-05a | 4.07E-06b | 5.51E-06ab | 3.12E-06 | lactose/L-arabinose transport system permease protein |
|  | K10191 | 1.02E-05a | 9.14E-06a | 9.87E-06a | 8.21E-06 | lactose/L-arabinose transport system ATP-binding protein |
|  | K10546 | 4.25E-05a | 2.32E-05b | 2.24E-05b | 1.64E-05 | putative multiple sugar transport system substrate-binding protein |
|  | K10547 | 4.71E-05a | 3.08E-05b | 2.69E-05b | 2.26E-05 | putative multiple sugar transport system permease protein |
|  | K10548 | 7.52E-05a | 5.02E-05b | 4.75E-05b | 3.58E-05 | putative multiple sugar transport system ATP-binding protein |
|  | K02025 | 6.72E-04b | 8.44E-04a | 8.32E-04a | 8.53E-04 | multiple sugar transport system permease protein |
|  | K02026 | 6.25E-04b | 7.61E-04a | 7.45E-04a | 7.68E-04 | multiple sugar transport system permease protein |
|  | K02027 | 9.74E-04b | 1.22E-03a | 1.22E-03a | 1.24E-03 | multiple sugar transport system substrate-binding protein |
|  | K10543 | 1.32E-04b | 1.59E-04a | 1.49E-04ab | 1.66E-04 | D-xylose transport system substrate-binding protein |
|  | K10544 | 1.31E-04a | 1.54E-04a | 1.53E-04a | 1.70E-04 | D-xylose transport system permease protein |
|  | K10545 | 1.15E-04a | 1.22E-04a | 1.15E-04a | 1.06E-04 | D-xylose transport system ATP-binding protein |
|  | K10552 | 4.23E-05a | 3.50E-05ab | 3.12E-05b | 2.88E-05 | fructose transport system substrate-binding protein |
|  | K10553 | 3.57E-05a | 2.71E-05a | 2.65E-05a | 2.65E-05 | fructose transport system permease protein |
|  | K10554 | 3.93E-05a | 3.10E-05a | 3.12E-05a | 2.94E-05 | fructose transport system ATP-binding protein |

Different lowercase letters represent significant differences among bamboo samples using Duncan's multiple range test. T test was used for comparing root zone soil of bamboo and spruce **p* < .05.BE: bamboo root endosphere, BR: bamboo rhizosphere, BZ: bamboo root zone, SZ: spruce root zone.

Table S6 CAZyme composition (classes) in the roots and soils.

| CAZyme class* | BE | BR | BZ | SZ |
| --- | --- | --- | --- | --- |
| AA | 0.82b | 3.08a | 3.06a | 3.13 |
| CBM | 4.37b | 19.39a | 19.42a | 19.16 |
| CE | 1.71b | 7.60a | 7.60a | 7.41 |
| GH | 33.80a | 31.57b | 31.46b | 31.31 |
| GT | 48.55a | 37.15b | 37.29b | 37.88 |
| PL | 10.76a | 1.21b | 1.19b | 1.11 |

Different lowercase letters represent significant differences among bamboo samples using Duncan's multiple range test. T test was used for comparing root zone soil of bamboo and spruce **p* < .05.BE: bamboo root endosphere, BR: bamboo rhizosphere, BZ: bamboo root zone, SZ: spruce root zone.

*. AA: Auxiliary Activities, refers to the redox enzymes that act in conjunction with CAZymes. CBM: Carbohydrate-Binding Modules. CE: Carbohydrate Esterases for hydrolysis of carbohydrate esters. GH: Glycoside Hydrolases for hydrolysis and/or rearrangement of glycosidic bonds. GT: Glycosyl Transferases responsible for the formation of glycosidic bonds. PL: Polysaccharide Lyases for non-hydrolytic cleavage of glycosidic bonds.

Table S7 CAZyme composition (families) in the roots and soils.

| CAZyme Family | BE | BR | BZ | SZ |
| --- | --- | --- | --- | --- |
| AA3 | 2.80E-03b | 1.37E-02a | 1.35E-02a | 1.32E-02 |
| AA7 | 9.66E-04b | 5.67E-03a | 5.67E-03a | 6.37E-03 |
| CBM13 | 3.91E-03b | 2.23E-02a | 2.22E-02a | 2.21E-02 |
| CBM2 | 5.40E-03b | 3.34E-02a | 3.35E-02a | 3.13E-02 |
| CBM32 | 2.49E-03b | 1.51E-02a | 1.50E-02a | 1.54E-02 |
| CBM47 | 2.12E-03b | 1.54E-02a | 1.54E-02a | 1.49E-02 |
| CBM48 | 2.15E-03b | 1.24E-02a | 1.23E-02a | 1.21E-02 |
| CBM50 | 5.35E-03b | 2.66E-02a | 2.68E-02a | 2.68E-02 |
| CBM51 | 2.19E-03b | 1.69E-02a | 1.71E-02a | 1.72E-02 |
| CBM57 | 1.22E-02a | 5.61E-03b | 5.67E-03b | 6.02E-02 |
| CBM6 | 1.44E-03b | 1.00E-02a | 1.00E-02a | 1.00E-02 |
| CE0 | 1.05E-03b | 7.20E-03a | 7.07E-03a | 6.93E-03 |
| CE1 | 1.49E-03b | 9.46E-03a | 9.45E-03a | 9.31E-03 |
| CE11 | 1.72E-03b | 1.09E-02a | 1.10E-02a | 1.05E-02 |
| CE14 | 2.28E-03b | 1.60E-02a | 1.62E-02a | 1.65E-02** |
| CE4 | 1.94E-03b | 1.19E-02a | 1.19E-02a | 1.17E-02 |
| CE9 | 1.13E-03b | 7.71E-03a | 7.81E-03a | 7.78E-03 |
| GH0 | 5.46E-03b | 4.27E-02a | 4.34E-02a | 4.42E-02 |
| GH13 | 4.98E-03b | 2.99E-02a | 3.02E-02a | 3.05E-02 |
| GH15 | 1.02E-03b | 7.18E-03a | 7.24E-03a | 7.81E-02 |
| GH16 | 1.88E-02a | 8.56E-03b | 8.40E-03b | 8.03E-03 |
| GH18 | 3.08E-03b | 6.75E-03a | 6.77E-03a | 6.95E-03 |
| GH2 | 2.49E-03b | 1.40E-02a | 1.38E-02a | 1.35E-02 |
| GH20 | 8.33E-04b | 4.93E-03a | 4.90E-03a | 4.89E-03 |
| GH23 | 2.68E-03b | 1.57E-02a | 1.56E-02a | 1.56E-02 |
| GH28 | 1.61E-01a | 9.38E-03b | 8.98E-03b | 8.53E-03 |
| GH3 | 4.28E-03b | 2.20E-02a | 2.19E-02a | 2.15E-02 |
| GH33 | 7.95E-04b | 5.63E-03a | 5.68E-03a | 6.65E-03 |
| GH38 | 8.80E-02a | 3.04E-03b | 2.84E-03b | 2.99E-03 |
| GH39 | 8.21E-04b | 4.68E-03a | 4.77E-03a | 5.01E-03 |
| GH4 | 1.26E-03b | 5.93E-03a | 5.57E-03a | 5.32E-03 |
| GH43 | 1.3E-03b | 7.22E-03a | 7.08E-03a | 6.64E-03 |
| GH5 | 1.12E-03b | 7.22E-03a | 7.08E-03a | 6.64E-03 |
| GH78 | 1.09E-03b | 5.96E-03a | 5.91E-03a | 5.84E-03 |
| GH94 | 1.28E-03b | 5.85E-03a | 5.84E-03a | 5.37E-03 |
| GT0 | 2.63E-03b | 9.15E-03a | 9.09E-03a | 9.32E-03 |
| GT1 | 1.42E-01a | 5.91E-03b | 5.38E-03b | 5.47E-03 |
| GT2 | 2.22E-02b | 1.32E-01a | 1.32E-01a | 1.35E-01 |
| GT28 | 1.50E-03b | 8.43E-03a | 8.40E-03a | 8.32E-03 |
| GT30 | 9.48E-04b | 5.41E-03a | 5.41E-03a | 5.27E-03 |
| GT35 | 1.78E-03b | 1.12E-03a | 1.14E-03a | 1.09E-03 |
| GT4 | 1.35E-02b | 8.48E-02a | 8.56E-02a | 8.79E-02 |
| GT41 | 5.05E-03b | 3.54E-02a | 3.64E-02a | 3.83E-02 |
| GT47 | 2.60E-01a | 3.40E-03b | 2.86E-03b | 2.92E-03 |
| GT48 | 1.95E-02a | 1.29E-04b | 8.55E-05b | 7.81E-05 |
| GT51 | 3.83E-03b | 2.45E-02a | 2.43E-02a | 2.36E-02 |
| GT83 | 1.03E-03b | 6.44E-03a | 6.54E-03a | 6.58E-03 |
| GT84 | 6.70E-04b | 5.23E-03a | 5.26E-03a | 4.81E-03 |
| GT9 | 1.51E-03b | 1.03E-02a | 1.05E-02a | 1.05E-02 |
| PL4 | 1.06E-01a | 1.32E-03b | 1.14E-03b | 1.07E-03 |

Different lowercase letters represent significant differences among bamboo samples using Duncan's multiple range test. T test was used for comparing root zone soil of bamboo and spruce **p* < .05.BE: bamboo root endosphere, BR: bamboo rhizosphere, BZ: bamboo root zone, SZ: spruce root zone.

Table S8 Correlations between CAZyme composition (families) and environmental factors of root zone soils.

| CAZyme Family | TN (mg/g) | TC (mg/g) | NH_4_^+^N (mg/kg) | NO_3_^-^N (mg/kg) | AP (mg/kg) | AK (mg/kg) | MBC  (mg C /kg) | MBN  (mg N /kg) | pH | EC（us/cm） | Urease（mg/g/24h) | Protease (ug/g/h | Cellulase (mg/kg/h) | Invertase (mg/kg/h) |
| --- | --- | --- | --- | --- | --- | --- | --- | --- | --- | --- | --- | --- | --- | --- |
| AA3 | -0.448 | -0.434 | -0.458 | -0.145 | -0.407 | -0.032 | -0.483* | -0.452 | -0.449 | -0.086 | -0.542* | -0.080 | -0.173 | -0.574* |
| AA7 | -0.512* | -0.487* | -0.510* | -0.164 | -0.424 | -0.12164 | -0.484* | -0.453 | -0.460 | -0.16286 | -0.537* | -0.107 | -0.23124 | -0.620** |
| CBM13 | -0.461 | -0.444 | -0.476* | -0.169 | -0.390 | -0.081 | -0.506* | -0.477* | -0.469 * | -0.125 | -0.537* | -0.066 | -0.187 | -0.565* |
| CBM2 | -0.504* | -0.497* | -0.534* | -0.264 | -0.363 | 0.041 | -0.390 | -0.268 | -0.283 | -0.093 | -0.403 | -0.171 | -0.234 | -0.498* |
| CBM32 | -0.453 | -0.429 | -0.462 | -0.147 | -0.392 | -0.088 | -0.511* | -0.495* | -0.493* | -0.133 | -0.556* | -0.043 | -0.185 | -0.550* |
| CBM47 | -0.499* | -0.488* | -0.525* | -0.250 | -0.371 | 0.004 | -0.416 | -0.304 | -0.319 | -0.103 | -0.409 | -0.126 | -0.234 | -0.489* |
| CBM48 | -0.493* | -0.476* | -0.504* | -0.185 | -0.388 | -0.006 | -0.454 | -0.385 | -0.384 | -0.097 | -0.487* | -0.117 | -0.194 | -0.541* |
| CBM50 | -0.473* | -0.453 | -0.477* | -0.151 | -0.375 | -0.013 | -0.485* | -0.437 | -0.415 | -0.082 | -0.522* | -0.095 | -0.169 | -0.549* |
| CBM51 | -0.514* | -0.499* | -0.532* | -0.244 | -0.374 | 0.022 | -0.401 | -0.285 | -0.310 | -0.105 | -0.413 | -0.144 | -0.244 | -0.500* |
| CBM57 | 0.379 | 0.352 | 0.367 | 0.084 | 0.340 | 0.028 | 0.533* | 0.526* | 0.493* | 0.076 | 0.563* | -0.013 | 0.125 | 0.572* |
| CBM6 | -0.487* | -0.472* | -0.502* | -0.210 | -0.372 | -0.021 | -0.466* | -0.392 | -0.393 | -0.104 | -0.487* | -0.104 | -0.215 | -0.540* |
| CE0 | -0.461 | -0.446 | -0.469* | -0.172 | -0.364 | 0.005 | -0.480* | -0.419 | -0.408 | -0.073 | -0.499* | -0.092 | -0.177 | -0.535* |
| CE1 | -0.463 | -0.448 | -0.473* | -0.162 | -0.386 | -0.037 | -0.495* | -0.455 | -0.440 | -0.096 | -0.517* | -0.085 | -0.166 | -0.557* |
| CE11 | -0.469* | -0.455 | -0.480* | -0.185 | -0.368 | 0.028 | -0.467* | -0.392 | -0.383 | -0.067 | -0.486* | -0.116 | -0.190 | -0.529* |
| CE14 | -0.476* | -0.455 | -0.482* | -0.159 | -0.387 | -0.051 | -0.495* | -0.456 | -0.449 | -0.114 | -0.533* | -0.085 | -0.187 | -0.561* |
| CE4 | -0.470* | -0.450 | -0.477* | -0.148 | -0.384 | -0.050 | -0.483* | -0.448 | -0.434 | -0.102 | -0.543* | -0.079 | -0.171 | -0.570* |
| CE9 | -0.483* | -0.463 | -0.485* | -0.169 | -0.383 | -0.034 | -0.485* | -0.438 | -0.431 | -0.103 | -0.529* | -0.092 | -0.189 | -0.563* |
| GH0 | -0.509* | -0.489* | -0.525* | -0.214 | -0.379 | -0.038 | -0.449 | -0.375 | -0.385 | -0.129 | -0.487* | -0.113 | -0.231 | -0.543* |
| GH13 | -0.478* | -0.453 | -0.474* | -0.128 | -0.398 | -0.047 | -0.492* | -0.464 | -0.447 | -0.107 | -0.536* | -0.075 | -0.149 | -0.548* |
| GH15 | -0.440 | -0.409 | -0.428 | -0.060 | -0.391 | -0.130 | -0.528* | -0.555* | -0.519* | -0.131 | -0.563* | -0.033 | -0.115 | -0.557* |
| GH16 | 0.473* | 0.447 | 0.489* | 0.174 | 0.385 | 0.092 | 0.511* | 0.485* | 0.483* | 0.155 | 0.556* | 0.057 | 0.198 | 0.532* |
| GH18 | -0.377 | -0.357 | -0.384 | -0.099 | -0.366 | -0.088 | -0.547* | -0.560* | -0.536* | -0.088 | -0.602** | -0.002 | -0.179 | -0.589** |
| GH2 | -0.397 | -0.375 | -0.398 | -0.103 | -0.362 | -0.055 | -0.546* | -0.549* | -0.509* | -0.084 | -0.564* | -0.019 | -0.133 | -0.537* |
| GH20 | -0.446 | -0.424 | -0.455 | -0.159 | -0.373 | -0.038 | -0.505* | -0.471* | -0.464 | -0.101 | -0.540 * | -0.064 | -0.200 | -0.551* |
| GH23 | -0.476* | -0.452 | -0.469* | -0.128 | -0.403 | -0.051 | -0.499* | -0.468* | -0.454 | -0.104 | -0.546 * | -0.068 | -0.158 | -0.562* |
| GH28 | 0.468* | 0.448 | 0.476* | 0.161 | 0.379 | 0.040 | 0.496* | 0.453 | 0.443 | 0.106 | 0.534* | 0.076 | 0.185 | 0.560* |
| GH3 | -0.436 | -0.415 | -0.442 | -0.134 | -0.382 | -0.065 | -0.526* | -0.514* | -0.485* | -0.102 | -0.564* | -0.053 | -0.157 | -0.558* |
| GH33 | -0.504* | -0.492* | -0.525* | -0.244 | -0.377 | -0.015 | -0.436 | -0.338 | -0.351 | -0.117 | -0.436 | -0.134 | -0.244 | -0.533* |
| GH38 | 0.471* | 0.453 | 0.482* | 0.164 | 0.381 | 0.045 | 0.489* | 0.451 | 0.443 | 0.107 | 0.527* | 0.089 | 0.188 | 0.556* |
| GH39 | -0.461 | -0.438 | -0.469* | -0.152 | -0.395 | -0.099 | -0.516* | -0.502* | -0.491* | -0.138 | -0.560* | -0.046 | -0.170 | -0.560* |
| GH4 | -0.427 | -0.422 | -0.449* | -0.129 | -0.339 | -0.081 | -0.464 | -0.458 | -0.409 | -0.070 | -0.542* | -0.062 | -0.122 | -0.616** |
| GH43 | -0.394 | -0.386 | -0.414 | -0.138 | -0.338 | 0.004 | -0.519* | -0.494* | -0.452 | -0.050 | -0.502* | -0.079 | -0.139 | -0.530* |
| GH5 | -0.427 | -0.414 | -0.439 | -0.141 | -0.379 | -0.037 | -0.521* | -0.495* | -0.472* | -0.081 | -0.530* | -0.076 | -0.168 | -0.559* |
| GH78 | -0.408 | -0.391 | -0.406 | -0.098 | -0.372 | -0.010 | -0.511* | -0.492* | -0.476* | -0.055 | -0.540* | -0.039 | -0.137 | -0.551* |
| GH94 | -0.451 | -0.449 | -0.471* | -0.197 | -0.332 | 0.077 | -0.419 | -0.319 | -0.293 | -0.004 | -0.422 | -0.156 | -0.189 | -0.500* |
| GT0 | -0.431 | -0.407 | -0.429 | -0.120 | -0.389 | -0.104 | -0.530 | -0.531* | -0.518* | -0.139 | -0.579* | -0.016 | -0.143 | -0.580* |
| GT1 | 0.475* | 0.459 | 0.487* | 0.166 | 0.395 | 0.050 | 0.491 | 0.454 | 0.440 | 0.104 | 0.519* | 0.097 | 0.180 | 0.553* |
| GT2 | -0.467* | -0.446 | -0.472* | -0.146 | -0.379 | -0.061 | -0.501* | -0.473* | -0.457 | -0.111 | -0.542* | -0.073 | -0.172 | -0.564* |
| GT28 | -0.459 | -0.435 | -0.454 | -0.108 | -0.377 | -0.055 | -0.517* | -0.504* | -0.466 | -0.094 | -0.548* | -0.078 | -0.131 | -0.548* |
| GT30 | -0.457 | -0.437 | -0.462 | -0.137 | -0.388 | -0.029 | -0.491* | -0.454 | -0.436 | -0.078 | -0.529* | -0.073 | -0.166 | -0.533* |
| GT35 | -0.498* | -0.483* | -0.512* | -0.213 | -0.371 | 0.031 | -0.420 | -0.324 | -0.337 | -0.083 | -0.461 | -0.130 | -0.224 | -0.534* |
| GT4 | -0.467* | -0.443 | -0.468* | -0.134 | -0.384 | -0.072 | -0.509* | -0.490* | -0.473* | -0.119 | -0.549* | -0.069 | -0.167 | -0.562* |
| GT41 | -0.494 | -0.470* | -0.511* | -0.211 | -0.380 | -0.075 | -0.477* | -0.424 | -0.441 | -0.155 | -0.539* | -0.072 | -0.234 | -0.548* |
| GT47 | 0.473* | 0.453 | 0.480* | 0.160 | 0.384 | 0.044 | 0.497* | 0.457 | 0.443 | 0.106 | 0.530* | 0.083 | 0.180 | 0.555* |
| GT48 | 0.453 | 0.432 | 0.461 | 0.137 | 0.366 | 0.027 | 0.485* | 0.445 | 0.432 | 0.085 | 0.528* | 0.064 | 0.183 | 0.563* |
| GT51 | -0.474* | -0.453 | -0.473* | -0.139 | -0.371 | -0.004 | -0.485* | -0.442 | -0.414 | -0.076 | -0.529* | -0.104 | -0.157 | -0.550* |
| GT83 | -0.486 | -0.459 | -0.489* | -0.155 | -0.392 | -0.049 | -0.489* | -0.445 | -0.432 | -0.118 | -0.535* | -0.063 | -0.162 | -0.522 |
| GT84 | -0.473* | -0.461 | -0.487* | -0.197 | -0.336 | 0.054 | -0.443 | -0.358 | -0.336 | -0.050 | -0.471* | -0.133 | -0.173 | -0.498 |
| GT9 | -0.455 | -0.430 | -0.453 | -0.137 | -0.378 | -0.036 | -0.509* | -0.479* | -0.472 | -0.103 | -0.546* | -0.059 | -0.171 | -0.540* |
| PL4 | 0.464 | 0.444 | 0.470* | 0.154 | 0.378 | 0.036 | 0.493* | 0.451 | 0.435 | 0.096 | 0.529* | 0.075 | 0.179 | 0.560* |
